# Multidetector Computed Tomography and Aortic Stenosis: The Emerging Potential of Bridging Morphology and Severity Grading

**DOI:** 10.3390/diagnostics15243233

**Published:** 2025-12-17

**Authors:** Gabriele Cordoni, Diana Di Paolantonio, Maria Teresa Savo, Dan Alexandru Cozac, Eleonora Lassandro, Martina Palmisano, Giulia Andolina, Giorgio De Conti, Julien Ternacle, Raffaella Motta, Valeria Pergola

**Affiliations:** 1 Cardiology Unit, Cardio-Thoracic-Vascular and Public Health Department, Padova University Hospital, 35128 Padova, Italy; gabriele.cordoni@studenti.unipd.it (G.C.); diana.dipaolantonio@studenti.unipd.it (D.D.P.); savomariateresa1@gmail.com (M.T.S.); eleonora.lassandro@studenti.unipd.it (E.L.); mariamartina.palmisano@studenti.unipd.it (M.P.); giulia.andolina@studenti.unipd.it (G.A.); giorgio.deconti@aopd.veneto.it (G.D.C.); valeria.pergola@gmail.com (V.P.); 2 Physiology Department, George Emil Palade University of Medicine, Pharmacy, Science, and Technology of Targu Mures, 540142 Targu Mures, Romania; 3 Haut-Leveque Cardiologic Hospital, Bordeaux University, 33600 Pessac, France; julien.ternacle@chu-bordeaux.fr; 4 Radiology Unit, Cardio-Thoracic-Vascular and Public Health Department, Padova University Hospital, 35128 Padova, Italy

**Keywords:** aortic valve stenosis, cardiac computed tomography, echocardiography, aortic valve area, hybrid aortic valve area, severity grading

## Abstract

**Background/Objectives**: Echocardiography is the reference standard for grading aortic stenosis (AS); however, it yields discordant severity estimates in up to 40% of patients. Multidetector computed tomography (MDCT)-derived methods for calculating aortic valve area (AVA) may improve diagnostic concordance, but their diagnostic performance, validation against invasive hemodynamics, and the influence of left ventricular outflow tract (LVOT) morphology on severity grading remain insufficiently investigated. **Methods**: We retrospectively analyzed 307 patients with normal-flow, high-gradient calcific AS who underwent echocardiography, MDCT, and cardiac catheterization. AVA was calculated using (1) echocardiographic LVOT diameter, (2) hybrid Doppler–MDCT planimetric LVOT area, and (3) corrected echocardiographic LVOT diameter (×1.13). Agreement, correlation, and diagnostic performance were assessed using Bland–Altman analysis, Pearson correlation, ROC analysis, and McNemar’s test. Subgroups defined by diagnostic concordance and MDCT-derived LVOT size were compared using ANOVA. **Results**: Hybrid AVA showed a strong correlation with echocardiographic AVA (r = 0.749, *p* < 0.001), with a mean difference of +0.11 ± 0.15 cm^2^. Both methods demonstrated similar relationships with invasive and non-invasive hemodynamic markers of AS severity. When combined with echocardiography, the hybrid method increased concordant classification of severe AS by 8%. In contrast, corrected AVA performed significantly worse, leading to more discordant classifications. LVOT size was significantly associated with variability in AVA and Doppler velocity index, independent of flow status. **Conclusions**: Hybrid MDCT-derived AVA provides diagnostic performance equivalent to echocardiography and improves concordance in selected patients. LVOT size influences key echocardiographic parameters and may warrant tailored diagnostic thresholds.

## 1. Introduction

Transthoracic echocardiography (TTE) is the recommended first-line imaging modality for assessing aortic stenosis (AS) in both European and American guidelines [1,2]. However, it is limited by interobserver variability, suboptimal acoustic windows, underestimation of left ventricular outflow tract (LVOT) diameter—especially in heavily calcified valves—and flow-dependent inconsistencies [3,4].

To improve diagnostic accuracy, guidelines endorse a multiparametric approach combining mean transvalvular gradient, peak aortic jet velocity, Doppler velocity index (DVI), and aortic valve area (AVA) [2,5]. Yet discordance between AVA and mean gradient is observed in up to 40% of severe AS cases, complicating clinical decision-making in intermediate-risk patients where accurate grading is essential for timing and mode of intervention [6,7].

Standard AVA calculation uses the continuity equation, assuming circular LVOT geometry and uniform laminar flow [8,9]. These assumptions often fail in clinical practice due to basal septal hypertrophy, annular calcification, or elliptical LVOT anatomy, leading to systematic underestimation [10,11]. Three-dimensional modalities, such as multidetector computed tomography (MDCT) and 3D echocardiography, provide accurate LVOT reconstructions independent of geometric assumptions [7,12,13]. Owing to its strong correlation with surgical measurements, MDCT is currently considered the reference standard for annular sizing before transcatheter aortic valve implantation (TAVI) [14]. Moreover, growing evidence supports its broader application throughout the spectrum of AS management [15,16,17].

A hybrid Doppler–MDCT approach replaces echocardiographic LVOT diameter with MDCT planimetric area in the continuity equation, potentially reducing geometric error. Some authors have proposed adjusted AVA thresholds (e.g., 1.2 cm^2^) for hybrid assessment, but systematic validation against invasive hemodynamics is lacking [18]. A fixed MDCT-derived correction factor (1.13) has been tested to adjust the echocardiographic LVOT diameter and mitigate systematic underestimation [19]. This adjustment has shown potential for reclassifying patients from severe to moderate AS, with improved prognostic discrimination.

The role of LVOT size and morphology in modulating echocardiographic parameters is also underexplored, despite evidence that anatomical variation can significantly influence AVA and DVI values [20]. No prior study has evaluated the relationship between MDCT-derived AVA estimates and invasively measured pressure gradients while accounting for LVOT morphology.

Accordingly, this study aimed to (1) compare hybrid and corrected AVA calculation methods with standard echocardiography for identifying concordant severe AS; (2) assess their correlation with non-invasive and invasive hemodynamic parameters; and (3) determine the influence of MDCT-derived LVOT size on AVA and DVI.

## 2. Materials and Methods

### 2.1. Study Population

This retrospective study included 351 consecutive patients with moderate or severe calcific aortic stenosis (AS), diagnosed according to current American and European valvular heart disease guidelines [2,5], who underwent Doppler echocardiography, MDCT, and invasive cardiac catheterization between June 2015 and October 2024 as part of preprocedural planning for transcatheter or surgical aortic valve replacement. Imaging and catheterization were performed within a median of 13 days (interquartile range [IQR]: 6–50).

Exclusion criteria included low flow status (indexed stroke volume < 35 mL/m^2^), mitral stenosis, moderate or greater aortic or mitral regurgitation, congenital heart disease, infective endocarditis, prior aortic valve intervention, dynamic LVOT obstruction, bicuspid or rheumatic aortic valve, age < 18 years, or refusal to participate.

Low-flow low-gradient AS phenotypes were excluded, since the primary objective of the study was to validate MDCT-based AVA estimation methods against non-invasive and invasive haemodynamic parameters.

After applying exclusion criteria, the final study cohort comprised 307 patients. Baseline demographics, cardiovascular risk factors, and comorbidities were extracted from electronic health records. The study was conducted in accordance with the Declaration of Helsinki and approved by the local Ethics Committee of Padua University Hospital (protocol CET-ACEV code 6316/AO/25, date of approval 15 May 2025). Informed consent was obtained from all subjects involved in the study.

### 2.2. Echocardiography Measurements

All patients underwent comprehensive TTE using commercially available scanners (Vivid 7 or Vivid E9, GE Healthcare, Chicago, IL, USA, or EPIQ 7C, Philips Medical Systems, Hamburg, Germany). Continuous-wave Doppler recordings of aortic flow were acquired from multiple acoustic windows (apical five-chamber, apical three-chamber, and right parasternal views). Transvalvular gradients were calculated using the simplified Bernoulli equation.

AVA was derived using the continuity equation, multiplying the LVOT cross-sectional area—measured in the parasternal long-axis view at the level of aortic cusp insertion during systole—by the LVOT velocity–time integral (VTI) obtained from the apical five-chamber view, with the sample volume placed ~5 mm proximal to the aortic valve and optimized for spectral alignment, averaging across multiple cardiac cycles in patients with atrial fibrillation (AF) or other arrhythmias.

Peak and mean gradients were measured from the highest-quality continuous-wave Doppler envelope. Stroke volume (SV) was calculated as LVOT area × LVOT VTI and indexed to body surface area (Stroke Volume index, SVi). DVI was calculated as the ratio of LVOT VTI to aortic valve VTI.

Variability due to suboptimal alignment or inadequate Doppler envelope tracing was minimized through the use of standardized protocols performed by experienced operators. Mitigation strategies included careful optimization of probe positioning, assessment from multiple acoustic windows, appropriate gain and filter settings, and averaging across multiple cardiac cycles in patients with AF.

Left ventricular ejection fraction (LVEF), end-diastolic diameter (LVEDD), and end-diastolic volume (EDV and indexed EDVi) were measured according to current echocardiographic guidelines [21,22].

Patients with a mean gradient > 40 mmHg and AVA < 1.0 cm^2^, or a mean gradient ≤ 40 mmHg and AVA ≥ 1.0 cm^2^, were classified as having concordant AS. All other patients were classified as having discordant AS.

### 2.3. Multidetector Computed Tomography (MDCT) Measurements

MDCT imaging was performed using a 320 × 0.5 mm detector scanner (Aquilion ONE VISION Edition, Canon Medical Systems, Ōtawara, Japan). Image acquisition was carried out during the diastolic phase of the cardiac cycle (~75% of the R–R interval) without administration of negative chronotropic agents. Slice thickness was 0.75 mm and post-processing analysis was performed by a single blinded operator using dedicated software (Aquarius iNtuition, version 4.4.13.P4, TeraRecon Inc., Foster City, CA, USA).

The LVOT area was measured by manual planimetry in a cross-sectional plane just below the basal insertion of the aortic cusps, using multiplanar reconstruction in three orthogonal planes [23]. The Eccentricity Index (EI) of the aortic annulus was calculated as the ratio of minimum to maximum diameter and used to stratify patients into “Low EI” (more elliptical LVOT) and “High EI” groups.

Hybrid AVA was calculated by entering the diastolic MDCT-derived LVOT area into the continuity equation (see Supplementary Figures S1 and S2). Corrected AVA was obtained after adjustment of the echocardiographic LVOT diameter using the previously reported correction factor of 1.13.

### 2.4. Invasive Heart Catheterization

At the time of coronary angiography, all patients underwent right and left heart catheterization as part of routine diagnostic evaluation and procedural planning, according to local protocol. All invasive procedures were clinically indicated and performed as part of standard care, with no interventions performed solely for research purposes, ensuring no additional invasive tests were performed beyond those required for patient care. This approach allowed for comprehensive hemodynamic assessment without the need for additional radiation or contrast media beyond that required for coronary angiography [24,25]. The peak-to-peak transvalvular gradient was obtained using the single-catheter left ventricular–aortic pullback technique. In the presence of atrial fibrillation or other arrhythmias, averaged measurements across several cardiac cycles were obtained. This approach was adopted to reduce beat-to-beat variability. Cardiac output (CO) was determined using the Fick method and subsequently indexed to body surface area (cardiac index, CI).

### 2.5. Statistical Analysis

Continuous variables are expressed as mean ± standard deviation (SD), and categorical variables as absolute counts and percentages, as needed. Between-group comparisons of continuous variables were performed using Student’s or Welch’s t-test, as appropriate according to Levene’s test. Comparisons between multiple groups were made using one-way ANOVA. Assumption of linearity was assessed by visual inspection of residual plots. Categorical variables were compared using the χ^2^ test or Fisher’s exact test, based on expected cell counts.

Agreement between AVA measurement techniques was assessed using Bland–Altman analysis, along with Pearson correlation and linear regression. Comparisons of AVA measurement methods were performed by evaluating their correlations with non-invasive and invasive hemodynamic parameters; correlation analyses employed Pearson’s coefficient and both univariable and multivariable linear regression models. The Williams test was used to compare correlation coefficients. The diagnostic performance of MDCT-derived hybrid and corrected AVA in classifying severe AS—defined by concordance with mean transvalvular pressure gradient—was evaluated using McNemar’s test and receiver operating characteristic (ROC) curves; comparison between ROC curves was performed Via DeLong test. All statistical tests were two-tailed, with a significance threshold of *p* < 0.05. Analyses were conducted using Python v3.13.2 (Python Software Foundation, Wilmington, DE, USA, https://www.python.org, accessed on 5 March 2025) with the Pandas, NumPy, SciPy, Statsmodels, and Lifelines libraries.

Given that the corrected AVA is a deterministic transformation of the standard echocardiographic AVA, it was excluded from correlation and ROC analyses.

## 3. Results

### 3.1. Baseline Characteristics

Baseline age, sex, Body Mass Index (BMI), Body Surface Area (BSA), and prevalence of risk factors and comorbidities such as hypertension, dyslipidemia, smoking habit, diabetes, and coronary artery disease (CAD) are shown in Table 1.

Parameters were analyzed both in the overall study population and after stratification based on the agreement between the mean gradient and AVA. Table 2 presents echocardiographic characteristics for the overall cohort and for concordant and discordant AS subgroups. Severity distribution, based on mean gradient, is shown alongside morphological parameters reflecting valve-related impact, including left ventricular diameters, volumes, and ejection fraction. Table 3 reports MDCT-derived parameters, including LVOT dimensions, planimetric area, and eccentricity index, as well as invasive hemodynamic data from cardiac catheterization, such as peak-to-peak transvalvular gradient and cardiac index.

### 3.2. Echocardiographic Versus MDCT-Derived AVA

**Figure 1:**
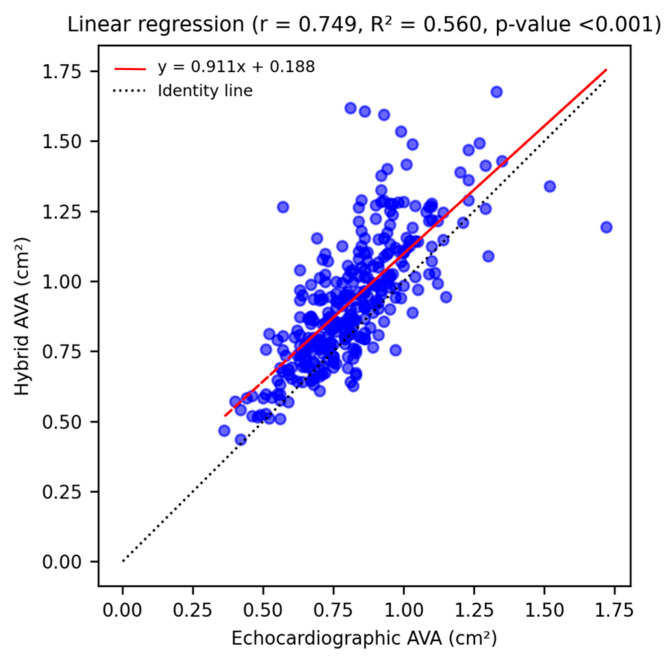
Linear univariate regression analysis shows a strong, statistically significant correlation (r = 0.749, *p*-value < 0.001) between echocardiographic and hybrid AVA measurements, and illustrates the tendency of MDCT-derived values to be larger than echocardiographic ones (red regression line lies above the identity line).

**Figure 2:**
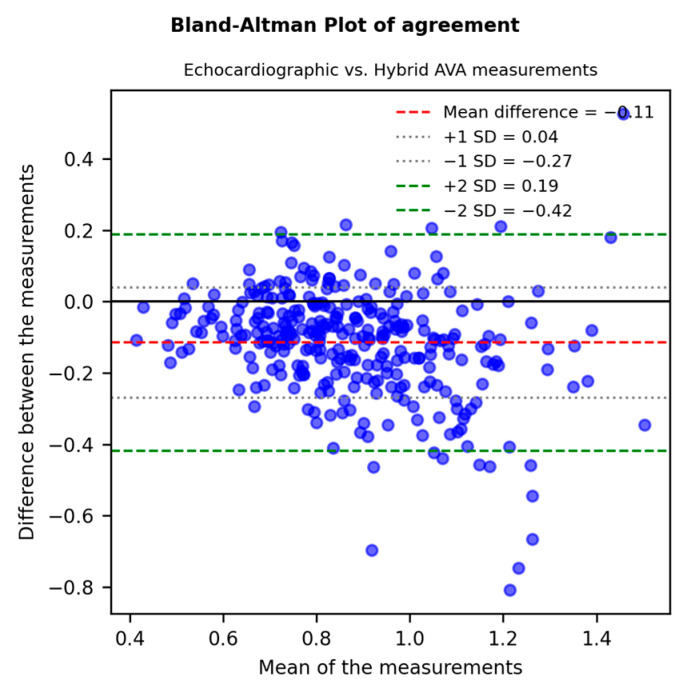
A Bland–Altman Plot (difference plot) assessing the agreement between echocardiographic and MDCT-derived “hybrid” AVA: echocardiographic AVA was significantly smaller with a mean difference of −0.11 ± 0.15 cm^2^ and a *p*-value < 0.001.

A strong and significant correlation between echocardiographic and hybrid measurements (r = 0.749, *p*-value < 0.001) was shown by linear univariate regression analysis (Figure 1). The agreement between measurements was assessed through a Bland–Altman Plot (Figure 2): echocardiographic AVA was significantly smaller with a mean difference of −0.11 ± 0.15 cm^2^.

### 3.3. Correlations with Non-Invasive and Invasive Aortic Stenosis Parameters

**Figure 3:**
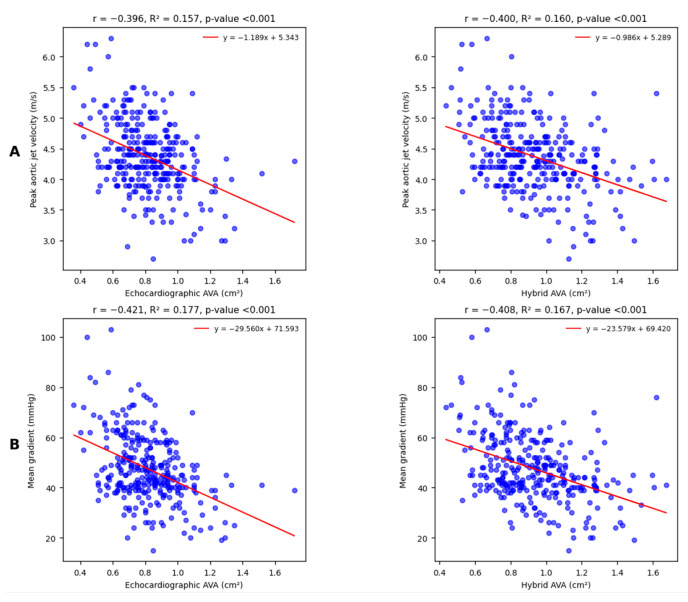
Linear univariate regression analysis shows moderate and statistically significant correlations with peak aortic jet velocity (**A**) and mean transvalvular gradient (**B**) for both echocardiographic and hybrid AVA. The Williams test suggests that the observed differences between correlation coefficients are not relevant (respectively, *p* = 0.930 and *p* = 0.733).

Firstly, echocardiographic and hybrid AVA were both equally and significantly correlated with the peak aortic jet velocity (Figure 3A) and the mean transvalvular gradient (Figure 3B).

**Figure 4:**
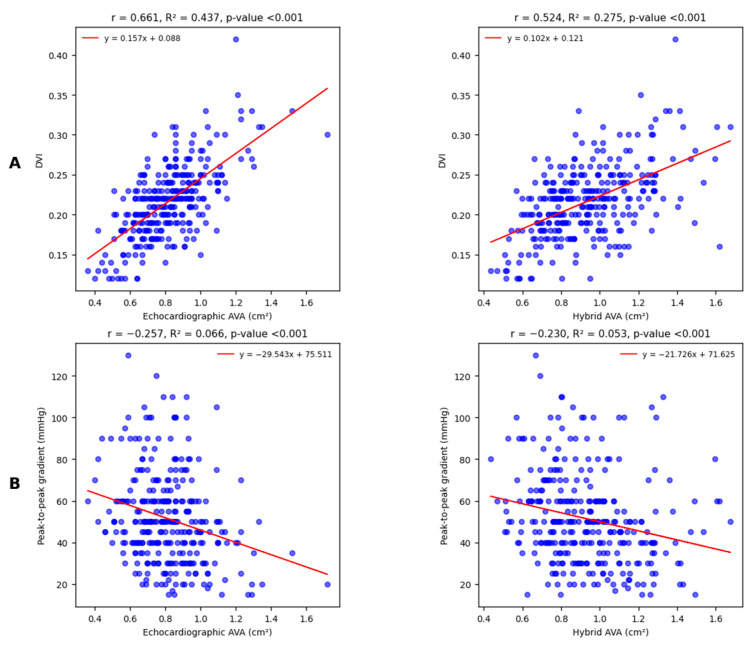
Linear univariate regression analysis shows moderate and statistically significant correlations with DVI (**A**); the observed difference between correlation coefficients is highly significant (Williams test *p* < 0.001). Conversely, regression analysis for peak-to-peak gradient (**B**) shows weak but statistically significant correlations for both echocardiographic and hybrid AVA. The observed difference between correlation coefficients is not relevant (Williams test *p* = 0.491).

Linear correlations between both types of AVA measurements and DVI appeared again moderate and statistically significant; however, in this case, echocardiographic AVA correlated to DVI in a slight but significantly stronger way (Figure 4A).

The last comparison involves a cardiac catheterization-derived parameter, the peak-to-peak gradient (Figure 4B): correlations are equally weak but significant.

Stratification into a “Low EI” subpopulation (EI ≤ 0.74, based on the mean displayed in Table 3) and a “High EI” group (EI ≥ 0.74) resulted in slight, non-significant differences in the correlations between hybrid AVA and either the mean or the peak-to-peak gradient, as shown in Supplementary Figure S3.

In addition, correlations between MDCT-derived AVA and pressure gradients were also assessed through multivariate linear regression analysis; after correction for identified confounding factors (such as SVi, BSA, and AF), hybrid AVA measurements remained significantly related to mean and peak-to-peak gradient values (*p-*value < 0.001).

### 3.4. Hybrid AVA as a Diagnostic Marker

**Figure 5:**
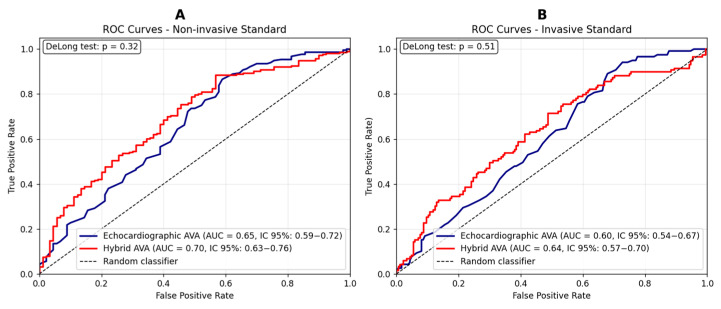
(**A**) ROC curves comparing hybrid and echocardiographic valvular surface values for the prediction of severe aortic stenosis, using the mean gradient as the reference standard. Sensitivity and specificity for the echocardiographic method: 0.87 and 0.41, respectively. Sensitivity and specificity for the hybrid method: 0.88 and 0.43, respectively. (**B**) ROC curves comparing hybrid and echocardiographic valvular surface values for the prediction of severe aortic stenosis, using the peak-to-peak gradient as the reference standard (>50 mmHg) [26]. Sensitivity and specificity for the echocardiographic method: 0.89 and 0.32, respectively. Sensitivity and specificity for the hybrid method: 0.71 and 0.52, respectively. A DeLong test *p*-value > 0.05 indicates that the observed differences between ROC curves are not statistically significant.

The hybrid AVA, used as a diagnostic marker of AS severity, demonstrated an AUC similar to that of the gold standard (echocardiographic AVA), based on its agreement with both non-invasive (Figure 5A) and invasive (Figure 5B) reference measures.

Its potential clinical value was assessed, given that invasive hemodynamic data are usually unavailable, by analyzing the concordance of both approaches with the mean gradient, defined as either a severely reduced AVA with a high mean gradient, or a non-severely reduced AVA with a low mean gradient (Table 4).

A comparable performance was observed, with no improvement in the reclassification of ambiguous AS cases into concordant diagnostic categories—nevertheless, the use of hybrid AVA in addition to the gold standard resulted in an 8% increase (*n* = 17) in concordant diagnoses.

Severity was defined according to current guideline-derived cutoffs: a mean gradient ≥ 40 mmHg and an echocardiographic aortic valve area (AVA) ≤ 1.0 cm^2^. Conversely, the previously proposed cutoff of 1.2 cm^2^ was adopted for the hybrid AVA [18], while a peak-to-peak gradient ≥ 50 mmHg was considered the conventionally accepted threshold [26]. In our normal-flow, high-gradient cohort, the mean transvalvular gradient represents the guideline-recommended primary classifier of severe AS and is routinely used in clinical practice; therefore, ROC analyses in Figure 5A were specifically designed to test whether the hybrid AVA is able to reproduce this gradient-based reference classification at least as well as standard echocardiographic AVA. Moreover, the correlation with the invasive peak-to-peak gradient in Figure 5B provides an additional and independent layer of validation. Atrial fibrillation was present in 35% of our study population, raising concerns about the inconsistency of transvalvular gradient measurements [27,28]. Supplementary Figures S4 and S5 display ROC curves stratified by cardiac rhythm, showing that hybrid AVA diagnostic performance was consistent between patients in sinus rhythm and those with atrial fibrillation.

On the contrary, the use of the corrected AVA through the LVOT diameter adjustment method appeared significantly inferior to echocardiographic AVA for agreement with mean gradient: the approach gold standard classified 71 more patients as concordant (*p*-value < 0.001, additional data are given in Supplementary Table S1).

### 3.5. MDCT for Morphological LVOT-Based Phenotyping

In our cohort, echocardiographic LVOT measurements were not associated with diagnostic inconsistencies, whereas MDCT-derived dimensions appeared to play a contributory role. To explore this further, patients were stratified into tertiles based on MDCT-derived LVOT area: small LVOT (≤33rd percentile, <3.8 cm^2^), average LVOT (33rd–66th percentile, 3.8–4.5 cm^2^), and large LVOT (≥66th percentile, >4.5 cm^2^). Parameters for each subgroup are summarized and compared in Table 5.

## 4. Discussion

The main results of our study are as follows: (1) the hybrid MDCT-derived AVA demonstrated a strong correlation with standard echocardiographic AVA (r = 0.749) and comparable diagnostic accuracy, while yielding slight overestimation (+0.11 cm^2^ on average); (2) when used in combination with echocardiography, the hybrid method increased concordant severe AS classifications by 8%; (3) the corrected AVA approach, based on a fixed MDCT-derived adjustment factor, performed significantly worse, producing more discordant classifications; and (4) LVOT size, assessed by MDCT planimetry, was significantly associated with variations in AVA and DVI, underscoring its potential role in refining severity thresholds.

Patients with discordant echocardiographic parameters were more frequently affected by moderate rather than severe AS, suggesting that such discrepancies tend to diminish as the disease progresses. This subgroup had more often lower body surface area, reduced LVEF, and a higher prevalence of atrial fibrillation—factors that can influence stroke volume and, consequently, the reliability of both mean gradient and continuity equation-derived AVA. Invasively measured cardiac index values were also lower in this group, with borderline statistical significance (*p*-value = 0.06).

LVOT size emerged as an additional determinant of diagnostic discrepancies. Consistent with previous findings [20,29], larger LVOT dimensions were associated with higher AVA and lower DVI values, both in a statistically and clinically relevant manner. Such variability can introduce misclassification when fixed diagnostic cutoffs are applied, regardless of anatomical differences and their impact on flow dynamics. While echocardiography did not detect significant LVOT size variation across subgroups, MDCT planimetry enabled accurate morphological phenotyping, linking structural characteristics to severity grading. Our data parallel the work of Michelena et al. [20], who demonstrated that small LVOT size is more common in women, with a smaller body size and lower cardiac output; however, in our cohort, differences in both AVA and invasively obtained CO were no longer statistically significant after BSA indexation.

Across LVOT size tertiles, peak velocity, transaortic gradients, and AVA tended to increase, whereas DVI decreased. Only AVA and DVI changes reached high statistical significance, representing 10–15% shifts in mean values across subgroups. These findings highlight the limitations of rigid application of universal thresholds for disease markers, as values can be influenced by anatomical and flow-related factors independent of valve pathology. Prospective studies stratifying outcomes by LVOT size are warranted to determine whether tailored severity thresholds would improve diagnostic accuracy and patient management. However, our cohort was not designed or powered to derive outcome-based, morphology-driven cut-offs, and such thresholds will require validation in larger and less selected populations before they can be incorporated into clinical decision algorithms or guidelines.

The hybrid method’s value was most apparent in anatomically challenging cases—poor acoustic windows, heavy annular calcification, and markedly elliptical LVOT geometry—where standard echocardiography is more prone to error. However, its performance did not exceed echocardiography overall, likely due to continued reliance on Doppler-derived VTI measurements, which remain operator-dependent and subject to variability [30,31]. From a hemodynamic standpoint, effective orifice area can differ from anatomical cross-sectional area because of heterogeneous velocity profiles and wall friction effects [18,32], challenging the assumption that geometric accuracy alone improves functional estimation.

The corrected AVA resulted in more discordant classifications, highlighting the pitfalls of a fixed correction factor when applied to anatomically diverse populations.

These observations are aligned with the growing emphasis on multimodality imaging in structural heart disease. Cardiac MRI, which does not involve radiation exposure, has limitations in the visualization of calcifications, and its use is less widespread because it is technically demanding and both time- and cost-consuming [31]. Three-dimensional transesophageal echocardiography has been shown to be comparable to MDCT for TAVR planning and does not require either contrast medium or radiation exposure, offering an economically attractive option that is particularly advantageous for patients with severe CKD [32].

Within this multimodality framework, our data support the role of MDCT-based hybrid AVA estimation as a pragmatic, targeted adjunct to transthoracic echocardiography in patients with discordant grading or challenging acoustic windows, rather than as a replacement for echocardiography in routine AS assessment. Our study highlights that, in borderline moderate-to-severe or discordant cases, integrating MDCT-derived anatomical data with Doppler flow assessment allows for a more comprehensive evaluation that can help confirm diagnostic concordance and reduce uncertainty; in this context, even small measurement inconsistencies may lead to misclassification.

From a practical standpoint, the hybrid AVA is best integrated as a targeted adjunct in patients with normal-flow, high-gradient AS and borderline or discordant echocardiographic findings, such as mean gradients close to the 40 mmHg threshold and AVA values only slightly below 1.0 cm^2^, particularly in the presence of suboptimal acoustic windows or atypical LVOT morphology. In these scenarios, the hybrid estimate can serve as an additional parameter to support or question diagnostic concordance and to reduce uncertainty before proceeding to invasive assessment or therapeutic decision-making. Conversely, in clearly severe or clearly moderate AS with high-quality echocardiographic data, the incremental value of hybrid AVA is expected to be limited. Importantly, this imaging technique provides superior spatial resolution without increasing costs or radiation exposure in our setting, as MDCT is already routinely performed for pre-procedural TAVR planning [14]. Advances in flow-independent imaging, computational fluid dynamics, and machine learning-driven segmentation hold promise for overcoming current limitations and improving reproducibility. Until such technologies are widely available, the hybrid method represents a pragmatic, targeted adjunct—bridging structural and functional domains—to enhance diagnostic confidence and guide management in selected patients with AS. Given the high prevalence of atrial fibrillation in the TAVR population, inclusion of AF patients enhances the real-world relevance of our findings and supports generalizability to broader clinical settings.

## 5. Limitations

This study has several limitations. First, it was conducted at a single tertiary centre and included only patients referred for aortic valve replacement. Our cohort therefore represents a highly selected population of patients with moderate-to-severe AS and specific clinical characteristics warranting surgical or transcatheter intervention, which introduces referral and selection bias and limits the generalizability of our findings. Second, MDCT measurements were obtained during diastole, whereas systolic imaging is generally preferred for procedural planning [14]. However, prior CT studies have shown only small, clinically negligible differences between systolic and diastolic LVOT area measurements [18], and have reported reduced motion artefacts and improved overall image quality with diastolic acquisition [33]. In our cohort, the systematic use of a diastolic phase for all MDCT-derived measurements makes it unlikely that timing-related bias materially affected LVOT or AVA estimates, or the head-to-head comparison between methods and their correlations with invasive hemodynamics. Third, planimetric AVA was not assessed, despite growing evidence of its prognostic relevance [33,34]. Fourth, the present study was not designed or powered to assess clinical outcomes, and our analyses are limited to diagnostic and haemodynamic validation; as a result, no conclusions can be drawn regarding the prognostic impact or management benefits of using hybrid AVA in routine practice. Fifth, we included patients with atrial fibrillation in line with prior studies demonstrating that continuity-equation AVA is feasible and reliable in this rhythm [27,28]. However, invasive pressure gradients in AF may be affected by beat-to-beat variability, and despite averaging multiple pullback recordings, some degree of inconsistency remains [35]. This limitation particularly affects the interpretation of invasive gradients in AF and should be considered when comparing diagnostic methods in this subgroup. Finally, our findings are limited to patients with normal-flow, high-gradient AS and may not be directly applicable to those with low-flow, low-gradient AS. Future investigations specifically designed for low-flow, low-gradient AS are warranted to determine whether our findings can be extended to this diagnostically challenging subgroup.

## 6. Conclusions

By enabling precise three-dimensional LVOT planimetry and accurate morphological phenotyping, MDCT addresses key limitations of ultrasound-based measurements, such as interobserver variability, poor acoustic windows, heavy annular calcification, and elliptical LVOT geometry.

Beyond its established role in TAVI planning, MDCT emerges as a robust adjunct for comprehensive AS evaluation, integrating anatomical accuracy with hemodynamic assessment. LVOT size, readily and reproducibly quantified by MDCT, proved to be a significant determinant of AVA, suggesting that severity thresholds might be optimized through individualized, morphology-driven cutoffs.

These findings support the inclusion of MDCT-based hybrid assessment in multimodal diagnostic algorithms, not only as a preprocedural tool, but also as a means to refine grading, improve concordance, and guide therapeutic decision-making. In the evolving landscape of structural heart imaging, MDCT is poised to play a central role in delivering more personalized and reproducible aortic stenosis evaluation.

## Figures and Tables

**Table 1:** Study cohort baseline demographic and clinical characteristics.

Mean ± SD or *n* (%)				
	Overall Population (*N* = 307)	Concordant AS Population (*N* = 237)	Discordant AS Population (*N* = 70)	*p*-Value ^1^
Age (years)	79 ± 7	79 ± 7	79 ± 7	0.94
Male sex	125 (40.7%)	88 (37.1%)	37 (52.9%)	**0.03**
BMI (kg/m^2^)	27.2 ± 5.0	27.7 ± 5.1	25.97 ± 4.47	**0.01**
BSA (m^2^)	1.8 ± 0.2	1.8 ± 0.2	1.79 ± 0.21	0.59
Diabetes	87 (28.3%)	65 (27.4%)	22 (31.4%)	0.62
Hypertension	266 (86.6%)	207 (87.3%)	58 (82.9%)	0.45
Dyslipidemia	223 (72.6%)	168 (70.9%)	55 (78.6%)	0.27
Smoking habit	81 (26.4%)	66 (27.8%)	14 (20.0%)	0.25
Family history of CAD	83 (27.0%)	64 (27.0%)	18 (25.7%)	0.95
AF	108 (35.2%)	71 (30.0%)	34 (48.6%)	**<0.01**
CAD	182 (59.3%)	135 (57.0%)	49 (70.0%)	0.07
CKD (eGFR < 30 mL/min/1.73 m^2^)	32 (10.4%)	20 (8.4%)	11 (15.7%)	0.12
Chest irradiation history	16 (5.2%)	15 (6.3%)	2 (2.9%)	0.41

^1^ Statistical significance of subgroups comparison. AF = Atrial Fibrillation; BMI = Body Mass Index; BSA = Body Surface Area; CAD = Coronary Artery Disease; CKD = Chronic Kidney Disease; eGFR = estimated Glomerular Filtration Rate. The bold values represent the statistically significant values.

**Table 2:** Study cohort baseline echocardiographic characteristics.

Mean ± SD or *n* (%)				
	Overall Population (*N* = 307)	Concordant AS Population (*N* = 237)	Discordant AS Population (*N* = 70)	*p*-Value ^1^
LVOT diameter (mm)	21.9 ± 1.8	21.8 ± 1.8	22.2 ± 2.0	0.16
LVOT derived area (cm^2^)	3.8 ± 0.6	3.8 ± 0.6	3.9 ± 0.7	0.14
LVEDD (mm)	45.9 ± 6.8	45.8 ± 6.8	46.4 ± 7.1	0.59
LVEDV (mL)	106.1 ± 37.4	105.5 ± 36.6	107.3 ± 40.1	0.72
LVEDVi (mL/m^2^)	58.7 ± 18.2	58.2 ± 17.6	60.0 ± 20.1	0.49
LVEF (%)	57.2 ± 9.9	58.4 ± 8.9	54.4 ± 11.8	**<0.01**
Peak aortic jet velocity (m/s)	4.4 ± 0.6	4.5 ± 0.5	4.0 ± 0.4	**<0.01**
Mean gradient (mmHg)	47.4 ± 13.3	50.3 ± 12.7	37.2 ± 8.2	**<0.01**
DVI	0.21 ± 0.05	0.21 ± 0.05	0.23 ± 0.04	**<0.01**
AVA (cm^2^)	0.8 ± 0.2	0.8 ± 0.2	0.9 ± 0.2	**<0.01**
AVA indexed (cm^2^/m^2^)	0.46 ± 0.10	0.45 ± 0.10	0.50 ± 0.10	**<0.01**
SV (mL)	86.0 ± 18.9	86.9 ± 17.6	83.3 ± 23.6	0.17
SVi (mL/m^2^)	47.6 ± 10.5	48.5 ± 9.7	46.8 ± 11.9	0.23
Moderate AS	91 (29.6%)	25 (10.5%)	49 (70.0%)	**<0.01**
Severe AS	216 (70.4%)	199 (84.0%)	17 (24.3%)	**<0.01**

^1^ statistical significance of subgroups comparison. DVI = Doppler Velocity Index; LVEDD = Left Ventricular End Diastolic Diameter; LVEDV = Left Ventricular End Diastolic Volume; LVEDVi = Left Ventricular End Diastolic Volume index; LVEF = Left Ventricular Ejection Fraction; SV = Stroke Volume; SVi = Stroke Volume index. The bold values represent the statistically significant values.

**Table 3:** Study cohort baseline MDCT and invasive characteristics.

Mean ± SD or *n* (%)				
	Overall Population (*N* = 307)	Concordant AS Population (*N* = 237)	Discordant AS Population (*N* = 70)	*p*-Value ^1^
LVOT diameter (mm)	23.2 ± 2.4	23.1 ± 2.4	23.8 ± 2.6	**0.03**
LVOT area (cm^2^)	4.3 ± 0.9	4.2 ± 0.9	4.5 ± 1.0	**0.03**
Corrected LVOT diameter (mm)	24.7 ± 2.1	24.7 ± 2.0	25.0 ± 2.3	0.20
Corrected LVOT area (cm^2^)	4.8 ± 0.8	4.8 ± 0.8	5.0 ± 0.9	0.18
LVOT EI	0.74 ± 0.08	0.73 ± 0.08	0.73 ± 0.07	0.89
Hybrid AVA (cm^2^)	0.93 ± 0.23	0.91 ± 0.23	1.02 ± 0.22	**<0.01**
Corrected AVA (cm^2^)	1.05 ± 0.25	1.04 ± 0.25	1.13 ± 0.25	**<0.01**
Peak-to-peak gradient (mmHg)	51.6 ± 22.6	54.9 ± 21.9	38.7 ± 16.6	**<0.01**
Cardiac output (L/min)	5.0 ± 1.2	5.1 ± 1.2	4.8 ± 1.1	0.8
Cardiac Index (L/min/m^2^)	2.8 ± 0.6	2.8 ± 0.6	2.7 ± 0.5	0.06

^1^ statistical significance of subgroups comparison. EI = Eccentricity Index. The bold values represent the statistically significant values.

**Table 4:** The McNemar test assessing the ability of the hybrid method to reclassify discordant aortic stenosis cases, based on the mean gradient.

	Discordant Diagnosis via Hybrid AVA	Concordant Diagnosis via Hybrid AVA
Discordant diagnosis via gold standard AVA	53	**17**
Concordant diagnosis via gold standard AVA	**27**	210
	*p*-value = 0.174	

The bold values represent discordant findings beween methods.

**Table 5:** Study cohort characteristics stratified according to LVOT area.

Mean ± SD or *n* (%)				
	Small LVOT Subgroup (*N* = 101)	Average LVOT Subgroup (*N* = 101)	Large LVOT Subgroup (*N* = 105)	*p*-Value ^1^
Discordant findings	15 (14.9%)	26 (25.7%)	29 (28.7%)	0.05
Age (years)	80.4 ± 5.6	78.8 ± 6.5	78.7 ± 7.7	0.13
Male sex	9 (8.9%)	35 (34.7%)	81 (77.1%)	**<0.01**
BSA (m^2^)	1.68 ± 0.17	1.78 ± 0.18	1.93 ± 0.20	**<0.01**
Peak aortic jet velocity (m/s)	4.5 ± 0.5	4.4 ± 0.6	4.3 ± 0.6	**0.03**
Mean gradient (mmHg)	49.1 ± 12.4	47.6 ± 14.2	45.1 ± 12.3	0.09
DVI	0.23 ± 0.05	0.22 ± 0.04	0.20 ± 0.04	**<0.01**
AVA (cm^2^)	0.77 ± 0.20	0.82 ± 0.19	0.86 ± 0.17	**<0.01**
AVAi (cm^2^/m^2^)	0.46 ± 0.11	0.46 ± 0.10	0.45 ± 0.09	0.74
Peak-to-peak gradient (mmHg)	55.0 ± 23.3	49.5 ± 20.9	49.2 ± 20.9	0.11
Cardiac output (L/min)	4.7 ± 1.3	5.1 ± 1.0	5.3 ± 1.3	**0.01**
Cardiac Index (L/min/m^2^)	2.83 ± 0.70	2.80 ± 0.46	2.73 ± 0.6	0.54

^1^ statistical significance of subgroups comparison. The bold values represent the statistically significant values.

## Data Availability

The raw data supporting the conclusions of this article will be made available by the authors on request.

## Supplementary Materials

The following supporting information can be downloaded at: https://www.mdpi.com/article/10.3390/diagnostics15243233/s1, Figure S1: planimetric LVOT measurement, Figure S2: echocardiographic and hybrid AVA calculation approaches, Figure S3: hybrid AVA correlations according to eccentricity index, Figure S4: ROC curves comparing hybrid and echocardiographic methods in the subpopulation with sinusal rhythm, Figure S5: ROC curves comparing hybrid and echocardiographic methods in the subpopulation with atrial fibrillation, Table S1: McNemar test assessing the ability of the correction factor to reclassify discordant aortic stenosis cases.

## Abbreviations

The following abbreviations are used in this manuscript:

AF	Atrial Fibrillation
AS	Aortic Stenosis
AVA	Aortic Valve Area
BSA	Body Surface Area
BMI	Body Mass Index
CAD	Coronary Artery Disease
CI	Cardiac Index
CKD	Chronic Kidney Disease
DVI	Doppler Velocity Index
eGFR	Estimated Glomerular Filtration Rate
EI	Eccentricity Index
LV	Left Ventricle / Left Ventricular
LVEDD	Left Ventricular End Diastolic Diameter
LVEDV	Left Ventricular End Diastolic Volume
LVEDVi	Left Ventricular End Diastolic Volume index
LVEF	Left Ventricular Ejection Fraction
LVOT	Left Ventricular Outflow Tract
MDCT	Multidetector Computed Tomography
ROC	Receiver Operating Characteristic
SV	Stroke Volume
SVi	Stroke Volume index
TTE	Transthoracic Echocardiography
VTI	Velocity–Time Integral
