# Supplementary material for: Multidetector Computed Tomography and Aortic Stenosis: The Emerging Potential of Bridging Morphology and Severity Grading
Source: Diagnostics (Basel). 2025 Dec 17;15(24):3233. doi: 10.3390/diagnostics15243233 (PMC12731794; doi:10.3390/diagnostics15243233)
Supplement: Supplementary file 1 [file diagnostics-15-03233-s001.zip › diagnostics-3972806-supplementary.pdf]

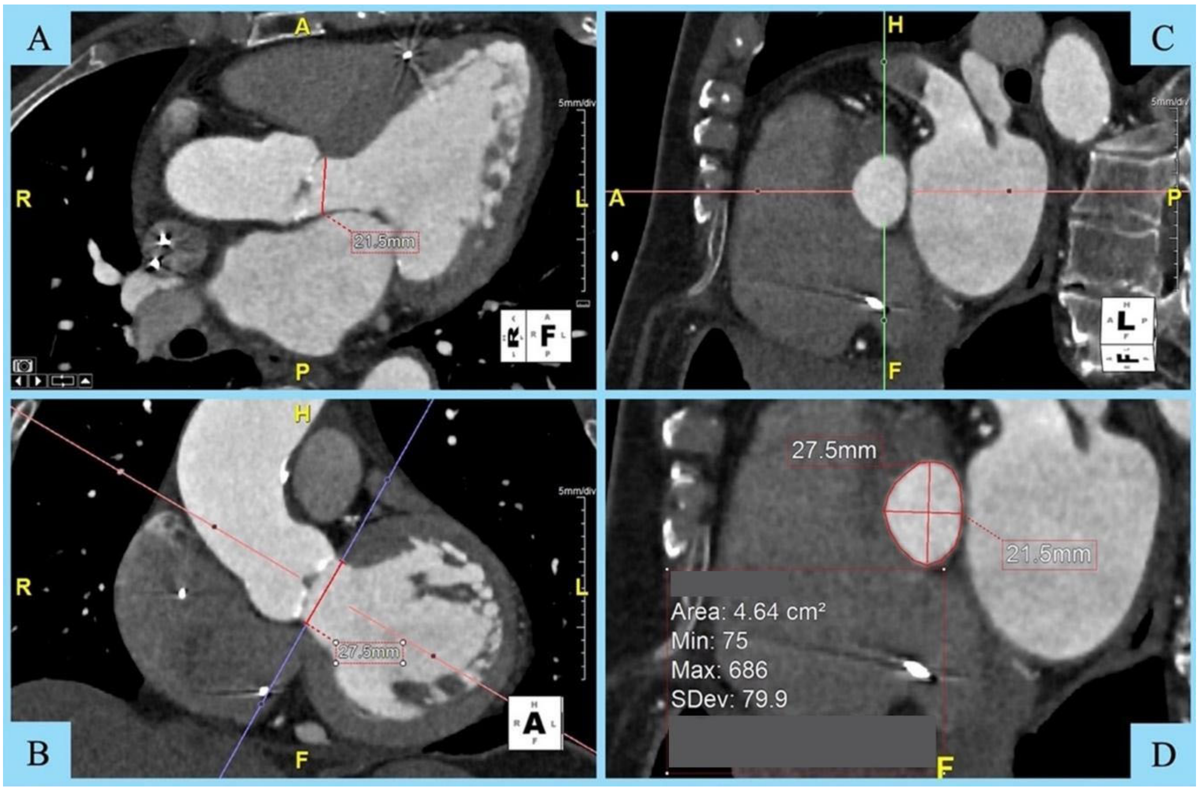

Supplementary Figure S1. *A, B) Two orthogonal planes: sagittal and coronal, respectively. C) Transverse section of the LVOT. D) Zoomed view of the LVOT with measurements of the diameters and planimetric area.*

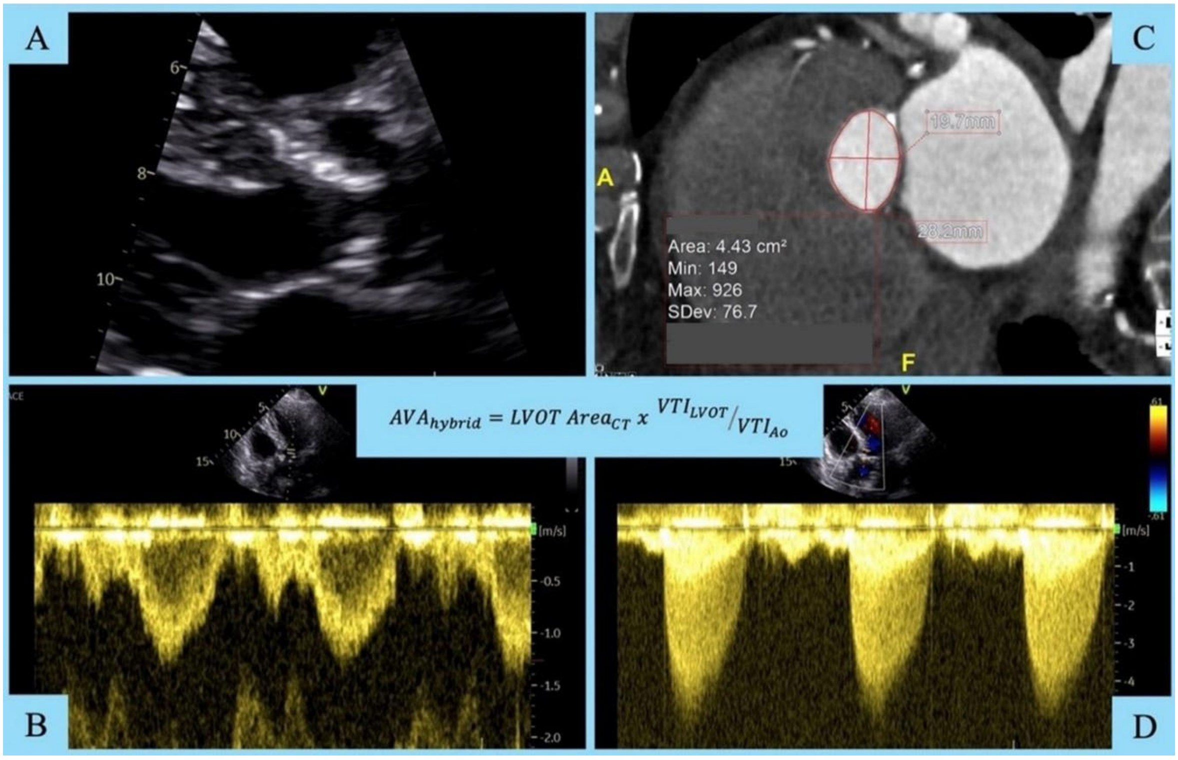

Supplementary Figure S2. *Continuity equation-derived echocardiographic and hybrid approaches for the calculation of AVA require**:***
A) Measurement of LVOT diameter by 2D transthoracic echocardiography from the PLAX view.
B) Planimetric assessment of LVOT area by cardiac MDCT.
C) Pulsed-wave Doppler in the LVOT from the apical 5-chamber (or apical 3-chamber) view to measure LVOT VTI.
D) Continuous-wave Doppler across the aortic valve from the apical 5-chamber (or apical 3-chamber) view to measure aortic VTI.

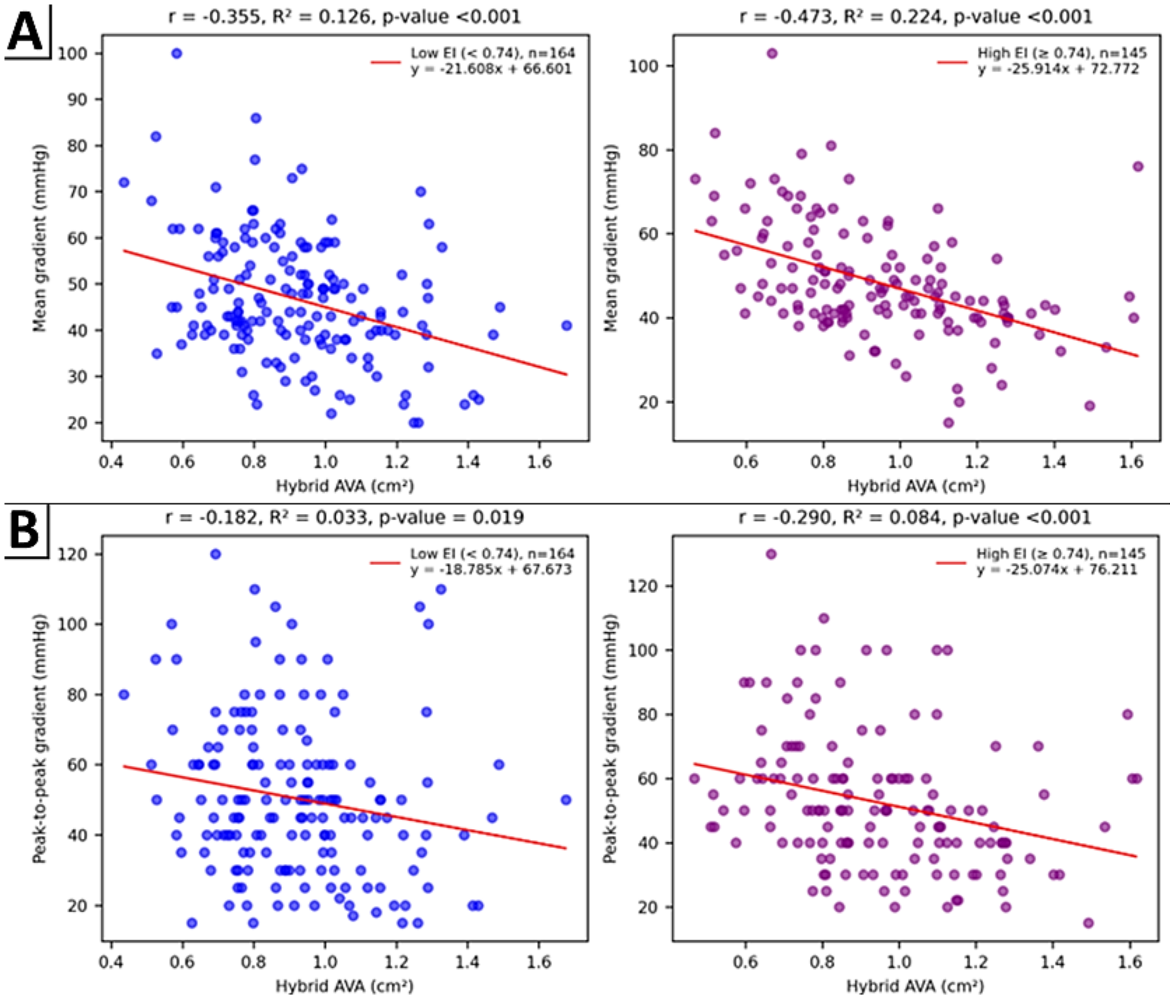

Supplementary Figure S3. Correlations between hybrid AVA measurements and both non-invasive (A) and invasive (B) gradient values assessed by univariate linear regression analysis, stratified according to LVOT Eccentricity Index (EI). Slight observed differences were judged statistically non-significant according to the Williams test (respectively, $p = 0.116$ and $p = 0.174$).

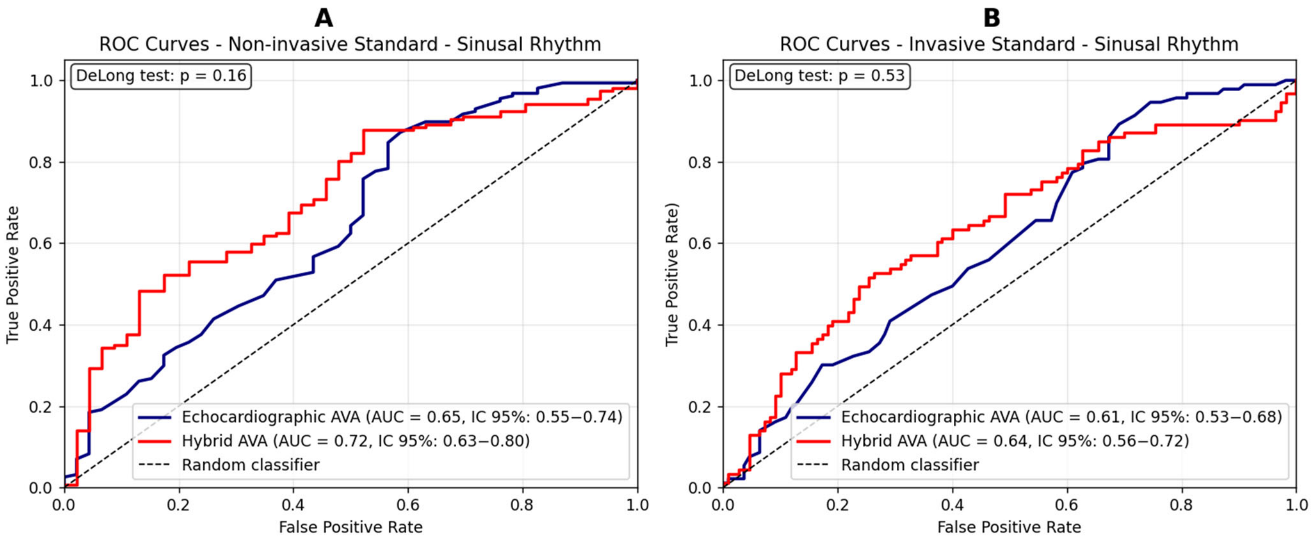

Supplementary Figure S4. (A) ROC curves comparing hybrid and echocardiographic valvular surface values for the prediction of severe aortic stenosis in the subpopulation with sinusal rhythm, using the mean gradient as the reference standard. Sensitivity and specificity for the echocardiographic method: 0.87 and 0.41, respectively. Sensitivity and specificity for the hybrid method: 0.88 and 0.48, respectively.
(B) ROC curves comparing hybrid and echocardiographic valvular surface values for the prediction of severe aortic stenosis in the subpopulation with sinusal rhythm, using the peak-to-peak gradient as the reference standard. Sensitivity and specificity for the echocardiographic method: 0.89 and 0.31, respectively. Sensitivity and specificity for the hybrid method: 0.52 and 0.74, respectively.

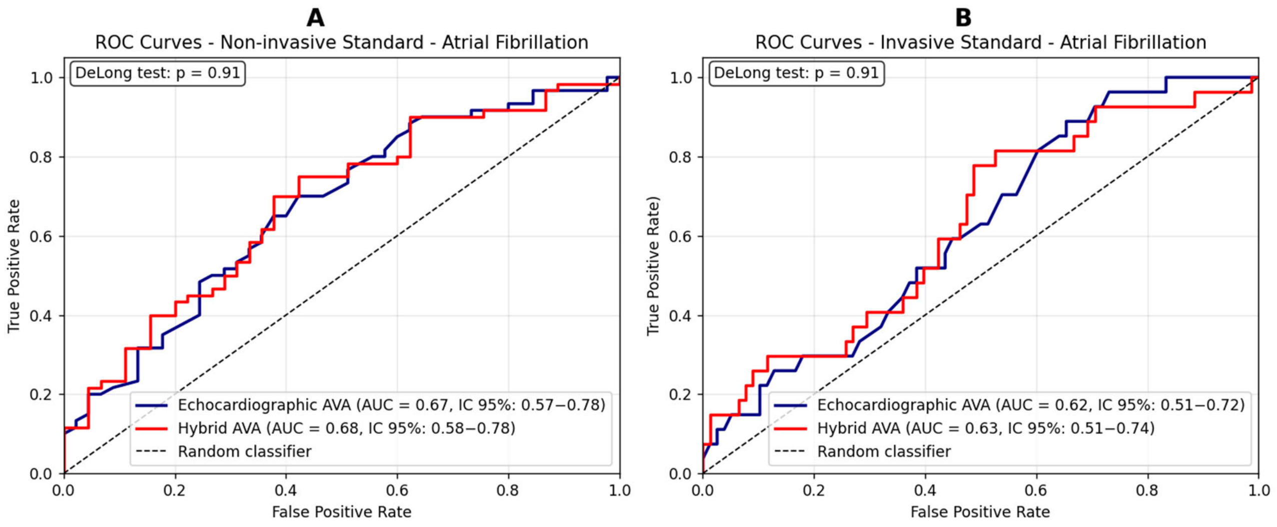

Supplementary Figure S5. (A) ROC curves comparing hybrid and echocardiographic valvular surface values for the prediction of severe aortic stenosis in the subpopulation with AF, using the mean gradient as the reference standard. Sensitivity and specificity for the echocardiographic method: 0.70 and 0.58, respectively. Sensitivity and specificity for the hybrid method: 0.75 and 0.58, respectively.
(B) ROC curves comparing hybrid and echocardiographic valvular surface values for the prediction of severe aortic stenosis in the subpopulation with AF, using the peak-to-peak gradient as the reference standard. Sensitivity and specificity for the echocardiographic method: 0.89 and 0.35, respectively. Sensitivity and specificity for the hybrid method: 0.78 and 0.51, respectively.

Supplementary Table S1. McNemar test assessing the ability of the correction factor to reclassify discordant aortic stenosis cases, based on mean gradient.

	Discordant diagnosis *via* corrected AVA	Concordant diagnosis *via* corrected AVA
Discordant diagnosis *via* gold standard AVA	42	**28**
Concordant diagnosis *via* gold standard AVA	**99**	138
	p-value < 0.001	
